# Supplementary material for: Signatures of soft selective sweeps predominate in the yellow fever mosquito Aedes aegypti
Source: bioRxiv. 2025 Jul 10:2025.07.06.663360. Preprint. [Version 1] doi: 10.1101/2025.07.06.663360 (PMC12265571; doi:10.1101/2025.07.06.663360)
Supplement: Supplement 5 [file media-5.pdf]

SUPPLEMENTAL DATA FOR:

Signatures of soft selective sweeps predominate in the yellow fever mosquito *Aedes aegypti*

Remi N. Ketchum[1], Daniel R. Matute[2], Daniel R. Schrider[1]

[1]Department of Genetics, University of North Carolina at Chapel Hill, Chapel Hill, North Carolina, 27514-2916, United States of America

[2]Department of Biology, University of North Carolina at Chapel Hill, Chapel Hill, North Carolina, 27514-2916, United States of America

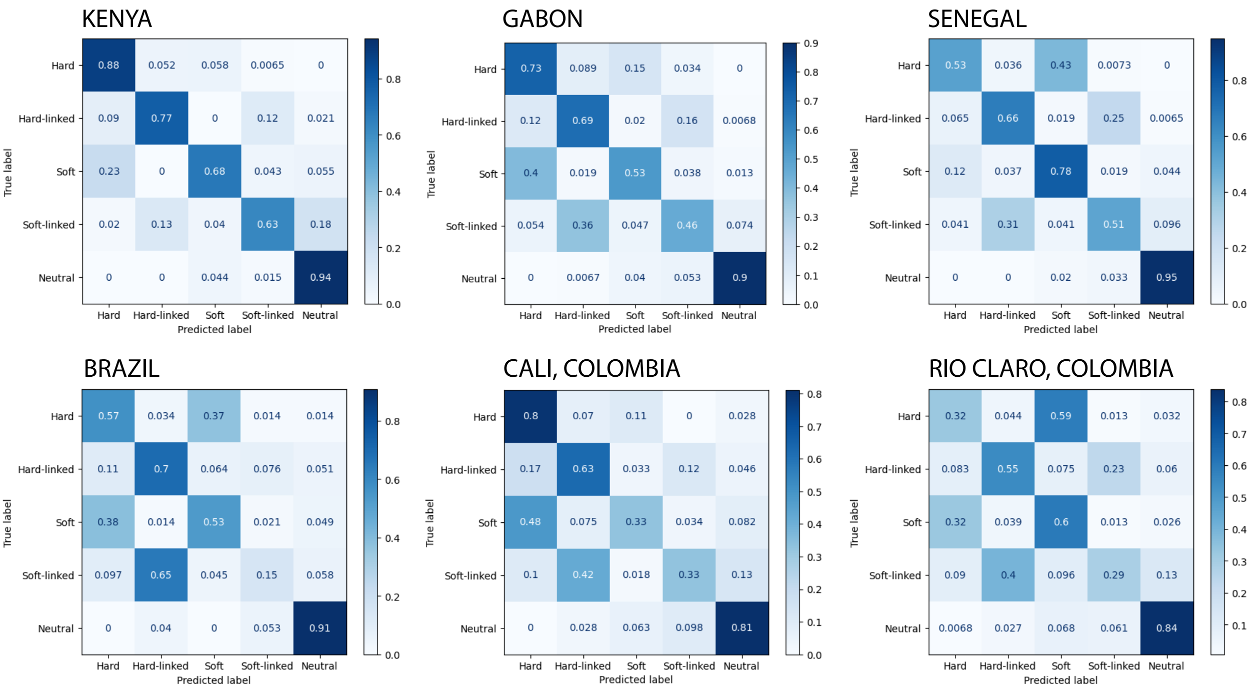

Supplemental Figure 1: Confusion matrices for each population sample with no posterior probability threshold applied.

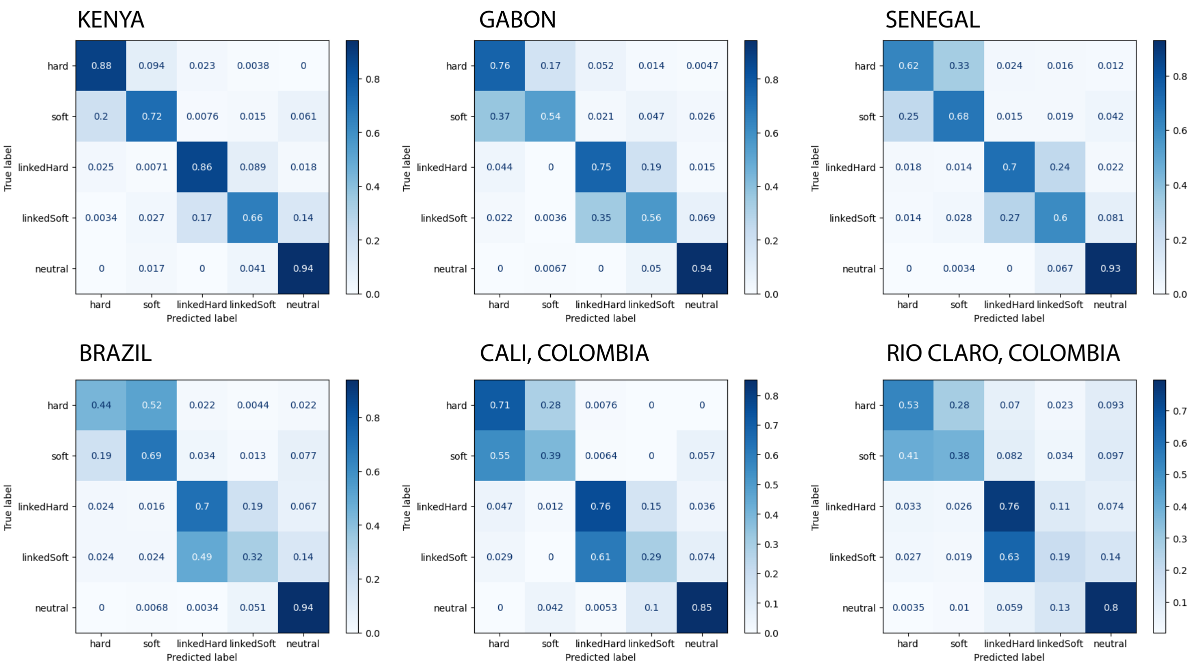

Supplemental Figure 2: Confusion matrices for each population sample with a 0.80 posterior probability threshold applied.

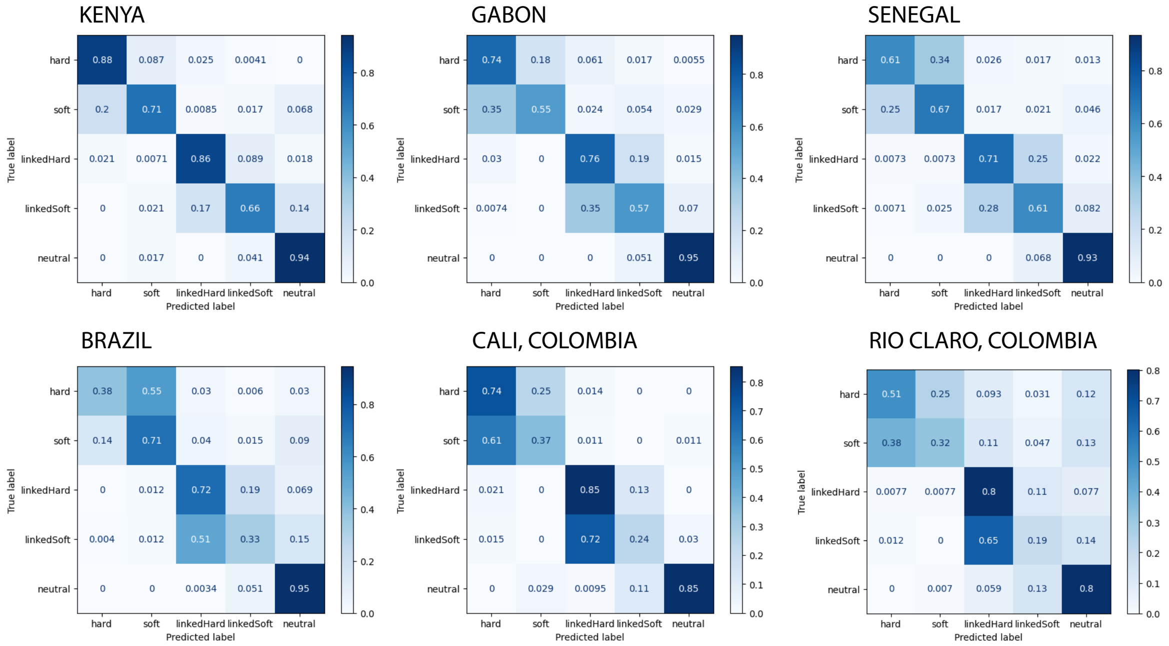

Supplemental Figure 3: Confusion matrices for each population sample with a 0.90 posterior probability threshold applied.

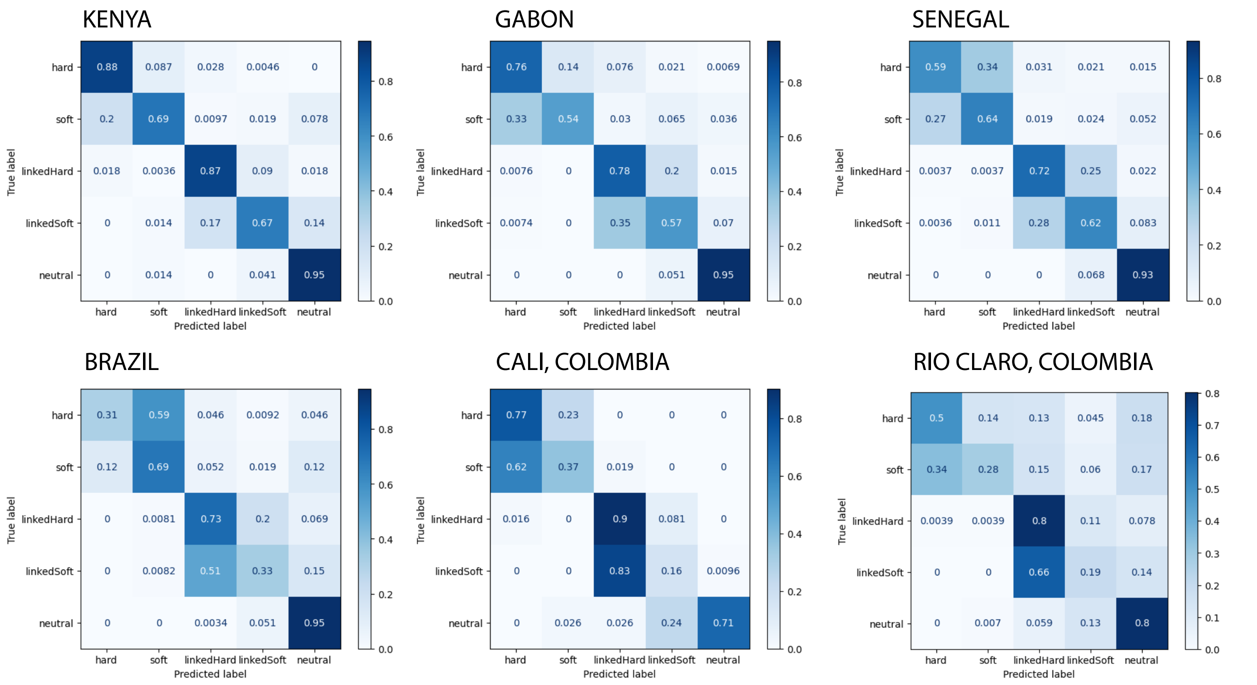

Supplemental Figure 4: Confusion matrices for each population sample with a 0.95 posterior probability threshold applied.

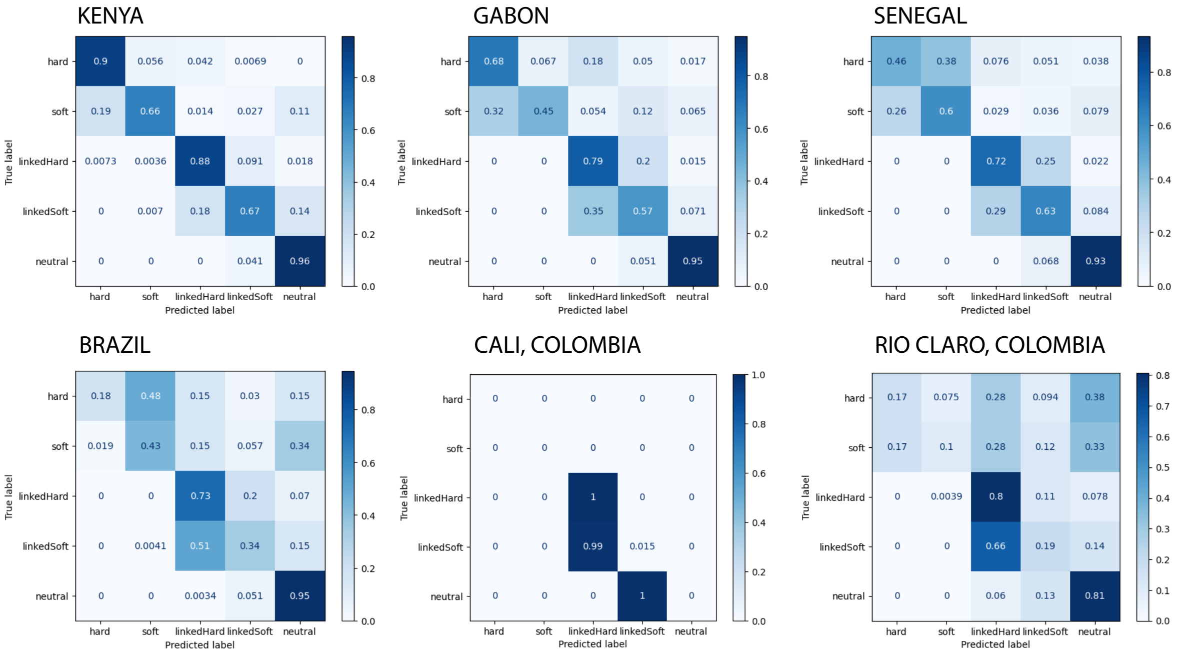

Supplemental Figure 5. Confusion matrices for each population sample with a 0.99 posterior probability threshold applied.

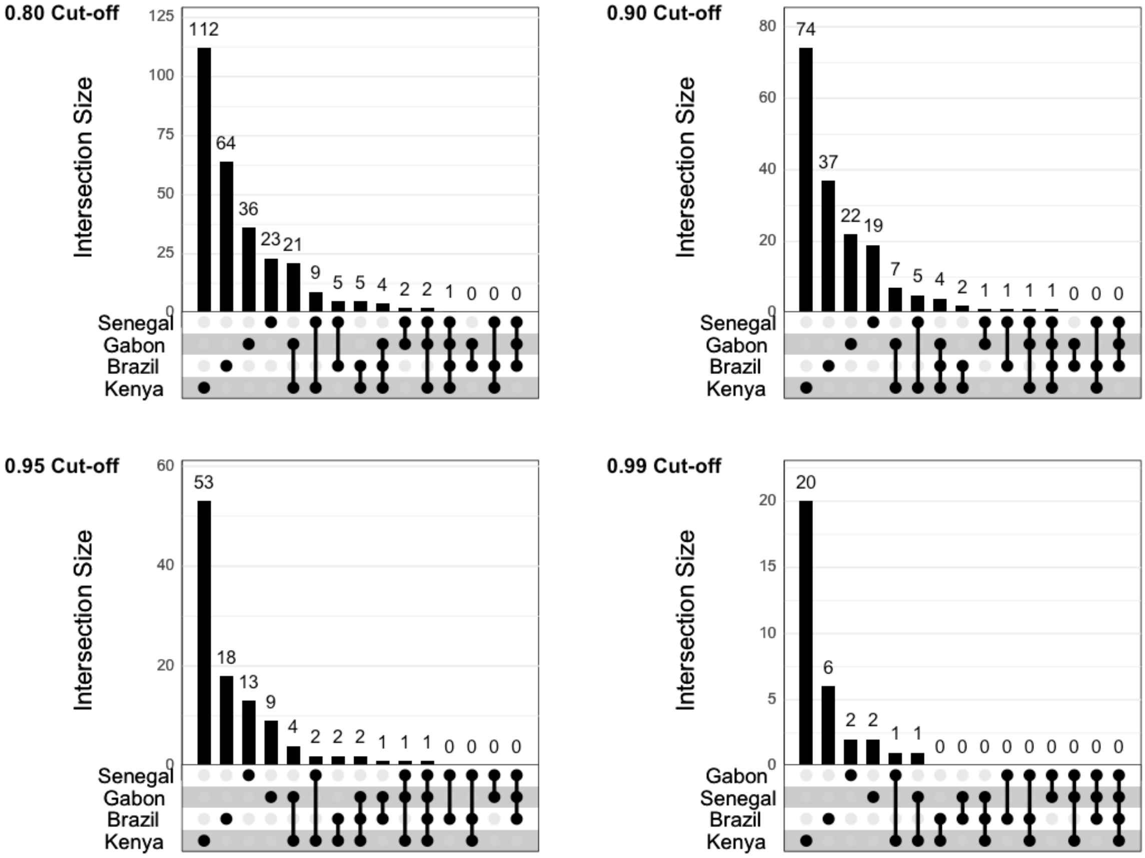

Supplemental Figure 6. Upset plots showing the overlap of sweeping windows discovered in diploS/HIC in each population sample at four posterior probability threshold cutoffs.

Supplemental Table 1: To evaluate our finding that soft sweeps predominate, we applied a conservative approach to estimate the proportion of soft sweeps after accounting for 1) hard sweeps that may be misclassified as soft and 2) potentially false positive soft sweeps. It should be noted that this approach is conservative because we do not account for the possibility that hard sweeps are misclassified as soft sweeps.

Supplemental Table 2: For each window classified as a sweep, we calculated the false discovery rate for a given threshold (based on the summed probabilities of a window being a soft sweep and hard sweep) by comparing the number of misclassified neutral simulations to the total number of predicted sweeps. We then calculated each window's associated *q*-values based on the combined posterior probabilities and our predicted false positive rates obtained from simulations. We did this for all windows classified as a sweep and for each population sample individually.

Supplemental Table 3: The 13 sweep windows that are shared between 2-4 population samples with a posterior probability cutoff ≥0.95. The table includes chromosome, window start and end, the populations where the sweep is present, the type of sweep classified, and the genes containing or overlapping the window.

Supplemental Table 4: The 93 sweep windows that are population-specific sweeps with a posterior probability cutoff ≥0.95. The table includes chromosome, window start and end, the population where the sweep is present, the type of sweep classified, and the genes containing or overlapping the window.

SUPPLEMENTARY TEXT

Population-Specific Sweep Windows Containing Well-Characterized IR Genes

We identified two high-confidence soft sweep windows in Kenya and one in Gabon that contained or overlapped three ATP-Binding Cassette (ABC) Transporter genes, ATP-binding cassette sub-family G member 4 (ABCG4; [chr2:455,500,001-455,750,000]), multidrug resistance-associated protein lethal (chr2:100,000,001-100,250,000) and multidrug resistance-associated protein 4 (chr3:138,250,001-138,500,000), respectively. In the first window, there is also a corresponding soft sweep in Brazil, although it did not meet the posterior probability cutoff at 0.86. Similarly, the second sweeping window also had a corresponding soft sweep in Gabon that did not meet the cutoff at 0.83. In humans, the multidrug resistant protein family (MRP) are transporters known for their role in causing multidrug resistance in tumor cells (Borst et al. 2000). In insects, ABC transporters have been documented as a group of detoxification-involved proteins (Labbé et al. 2011; Epis et al. 2014; Qi et al. 2016). In the mosquito *Culex pipiens pallens*, MDR1 and MDR2 were identified as proteins that were significantly enriched in cypermethrin resistance (Zhang et al. 2022) and in the Colorado potato beetle *Leptinotarsa decemlineata*, MRP4 was significantly upregulated in response to spinosa (Chen et al. 2023).

Population-Specific Sweeps Windows with Putative or Emerging IR Candidates

In Kenya, we found two sweeping windows that were classified as soft sweeps which overlapped or contained genes involved in the fatty acyl-CoA synthesis pathway, *acyl-CoA synthetase family member 4* (chr2:251,750,001-252,000,000) and *fatty acyl-CoA reductase 1* (chr3:309,250,001-309,500,000). *Acyl-CoA synthetase* (ACS), which activates fatty acids, was upregulated in the pea aphid (Cai et al. 2024) and was also found in a selection scan performed on *Anopheles gambiae*, along with several other IR genes (Dennis et al. 2024). It is also worth noting that this same window had a combined posterior probability of 0.94 in Senegal (marginally below our stringent 0.95 threshold). Fatty acyl-CoA reductases exhibit many biological functions, one of which being synthesis of cuticular hydrocarbons (CHCs; (Wang et al. 2024)). Many insecticides are absorbed through the insect cuticle and changes to the cuticle interface can slow the penetration of insecticides, thereby resulting in increased IR (Jacobs et al. 2023). In the cotton mealybug *Phenacoccus solenopsis*, a fatty acyl-CoA reductase (FAR) gene contributed to wax biosynthesis and RNA interference against *FAR* resulted in increased mortality post-deltamethrin treatments (Tong et al. 2022). Further, in a phosphine resistant strain of *Tribolium castaneum*, *FAR1* was upregulated and the cuticle structure was more continuous and tight than the susceptible strain (Kim et al. 2023).

We identified a window classified as a soft sweep in Gabon which contains the *membrane-associated progesterone receptor component 1* (chr1:15,500,001-15,750,000). This same window is classified as a soft sweep in Kenya and although the posterior probability of the window being a sweep (prob = 0.89) did not meet our cutoff, this may indicate a shared sweep between population samples. Membrane-associated progesterone receptor component 1 (PGRMC1) binds and stabilizes many different CYPs, thereby supporting P450 protein levels posttranscriptionally (McGuire et al. 2021). Further, when PGRMC1 was knocked out in mice livers, there was reduced enzyme levels of P450s. Although the role that PGRMC1 plays in insects is unclear, it was upregulated in response to imidacloprid exposure in honeybees (Kim et al. 2022).

In Kenya, we identified a sweeping window (chr1:48,750,001-49,000,000) that was classified as a soft sweep and overlapped the *D(2) dopamine receptor A*, which has been explored as new mode-of-action insecticide targets (Nuss et al. 2015). Further, pyrethroids are known to be potent releasers of dopamine (Elwan et al. 2006). In Brazil, we found a sweeping window that contains the *transcription factor grauzone* (chr1:63,750,001-64,000,000), is classified as a soft sweep, and contains a CLR peak > 540. The *transcription factor grauzone* is required for meiosis in oogenesis and it was significantly associated with resistance to pyrethroids in *Ae. aegypti* (Campbell et al. 2019) and *Anopeheles funestus* (Wondji et al. 2022).

In Brazil, we detected a window classified as a soft sweep which contained *NADH dehydrogenase 1 beta subcomplex subunit 5* (chr2:4,000,001-4,250,000). *NADH dehydrogenase 1 beta subcomplex subunit 5* encodes for a subunit of the mitochondrial NADH dehydrogenase (Complex 1) which has been shown to provide resistance to the toxicants paraquat and menadione in *D. melanogaster* (Gospodaryov et al. 2020). In another study, several subunits of the NADH dehydrogenase were upregulated in response to permethrin in *An. gambiae* which they attributed to a potential link between mitochondrial energy metabolism and detoxification (Vontas et al. 2005).

REFERENCES

Borst P, Evers R, Kool M, Wijnholds J. 2000. A family of drug transporters: the multidrug resistance-associated proteins. *Journal of the National Cancer Institute* 92:1295–1302.

Cai Z, Zhao X, Qian Y, Zhang K, Guo S, Kan Y, Wang Y, Ayra-Pardo C, Li D. 2024. Transcriptomic and Metatranscriptomic Analyses Provide New Insights into the Response of the Pea Aphid Acyrthosiphon pisum (Hemiptera: Aphididae) to Acetamiprid. *Insects* 15:274.

Campbell CL, Saavedra-Rodriguez K, Kubik TD, Lenhart A, Lozano-Fuentes S, Black IV WC. 2019. Vgsc-interacting proteins are genetically associated with pyrethroid resistance in Aedes aegypti. *PLoS One* 14:e0211497.

Chen YH, Cohen ZP, Bueno EM, Christensen BM, Schoville SD. 2023. Rapid evolution of insecticide resistance in the Colorado potato beetle, Leptinotarsa decemlineata. *Current Opinion in Insect Science* 55:101000.

Dennis TP, Essandoh J, Mable BK, Viana MS, Yawson AE, Weetman D. 2024. Signatures of adaptation at key insecticide resistance loci in Anopheles gambiae in Southern Ghana revealed by reduced-coverage WGS. *Scientific Reports* 14:8650.

Elwan MA, Richardson JR, Guillot TS, Caudle WM, Miller GW. 2006. Pyrethroid pesticide-induced alterations in dopamine transporter function. *Toxicology and Applied Pharmacology* 211:188–197.

Epis S, Porretta D, Mastrantonio V, Comandatore F, Sassera D, Rossi P, Cafarchia C, Otranto D, Favia G, Genchi C. 2014. ABC transporters are involved in defense against permethrin insecticide in the malaria vector Anopheles stephensi. *Parasites & Vectors* 7:1–7.

Gospodaryov DV, Strilbytska OM, Semaniuk UV, Perkhulyn NV, Rovenko BM, Yurkevych IS, Barata AG, Dick TP, Lushchak OV, Jacobs HT. 2020. Alternative NADH dehydrogenase extends lifespan and increases resistance to xenobiotics in Drosophila. *Biogerontology* 21:155–171.

Jacobs E, Chrissian C, Rankin-Turner S, Wear M, Camacho E, Broderick NA, McMeniman CJ, Stark RE, Casadevall A. 2023. Cuticular profiling of insecticide resistant Aedes aegypti. *Scientific Reports* 13:10154.

Kim D, Kim K, Lee YH, Lee S-E. 2023. Transcriptome and Micro-CT analysis unravels the cuticle modification in phosphine-resistant stored grain insect pest, Tribolium castaneum (Herbst). *Chemical and Biological Technologies in Agriculture* 10:88.

Kim S, Cho S, Lee SH. 2022. Synergistic effects of imidacloprid and high temperature on honey bee colonies. *Apidologie* 53:67.

Labbé R, Caveney S, Donly C. 2011. Expression of multidrug resistance proteins is localized principally to the Malpighian tubules in larvae of the cabbage looper moth, Trichoplusia ni. *Journal of Experimental Biology* 214:937–944.

McGuire MR, Mukhopadhyay D, Myers SL, Mosher EP, Brookheart RT, Kammers K, Sehgal A, Selen ES, Wolfgang MJ, Bumpus NN. 2021. Progesterone receptor membrane component 1 (PGRMC1) binds and stabilizes cytochromes P450 through a heme-independent mechanism. *Journal of Biological Chemistry* 297:101316.

Nuss AB, Ejendal KF, Doyle TB, Meyer JM, Lang EG, Watts VJ, Hill CA. 2015. Dopamine receptor antagonists as new mode-of-action insecticide leads for control of Aedes and Culex mosquito vectors. *PLoS Neglected Tropical Diseases* 9:e0003515.

Qi W, Ma X, He W, Chen W, Zou M, Gurr GM, Vasseur L, You M. 2016. Characterization and expression profiling of ATP-binding cassette transporter genes in the diamondback moth, Plutella xylostella (L.). *BMC Genomics* 17:1–18.

Tong H, Wang Yuan, Wang S, Omar MA, Li Zicheng, Li Zihao, Ding S, Ao Y, Wang Ying, Li F. 2022. Fatty acyl-CoA reductase influences wax biosynthesis in the cotton mealybug, Phenacoccus solenopsis Tinsley. *Communications Biology* 5:1108.

Vontas J, Blass C, Koutsos AC, David J-P, Kafatos FC, Louis C, Hemingway J, Christophides GK, Ranson H. 2005. Gene expression in insecticide resistant and susceptible Anopheles gambiae strains constitutively or after insecticide exposure. *Insect Molecular Biology* 14:509–521.

Wang Z, Andika IP, Chung H. 2024. Regulation of insect cuticular hydrocarbon biosynthesis. *Current Opinion in Insect Science*:101287.

Wondji CS, Hearn J, Irving H, Wondji MJ, Weedall G. 2022. RNAseq-based gene expression profiling of the Anopheles funestus pyrethroid-resistant strain FUMOZ highlights the predominant role of the duplicated CYP6P9a/b cytochrome P450s. *G3* 12:jkab352.

Zhang C, Guo X, Li T, Cheng P, Gong M. 2022. New insights into cypermethrin insecticide resistance mechanisms of Culex pipiens pallens by proteome analysis. *Pest Management Science* 78:4579–4588.
